# Jargon and Readability in Plain Language Summaries of Health Research: Cross-Sectional Observational Study

**DOI:** 10.2196/50862

**Published:** 2025-01-13

**Authors:** Iain A Lang, Angela King, Kate Boddy, Ken Stein, Lauren Asare, Jo Day, Kristin Liabo

**Affiliations:** 1 Department of Health and Community Sciences University of Exeter Medical School University of Exeter Exeter United Kingdom

**Keywords:** readability, jargon, reading, accessibility, health research, science communication, public understanding of science, open science, patient and public involvement, health literacy, plain language summary, health communication

## Abstract

**Background:**

The idea of making science more accessible to nonscientists has prompted health researchers to involve patients and the public more actively in their research. This sometimes involves writing a plain language summary (PLS), a short summary intended to make research findings accessible to nonspecialists. However, whether PLSs satisfy the basic requirements of accessible language is unclear.

**Objective:**

We aimed to assess the readability and level of jargon in the PLSs of research funded by the largest national clinical research funder in Europe, the United Kingdom’s National Institute for Health and Care Research (NIHR). We also aimed to assess whether readability and jargon were influenced by internal and external characteristics of research projects.

**Methods:**

We downloaded the PLSs of all NIHR National Journals Library reports from mid-2014 to mid-2022 (N=1241) and analyzed them using the Flesch Reading Ease (FRE) formula and a jargon calculator (the De-Jargonizer). In our analysis, we included the following study characteristics of each PLS: research topic, funding program, project size, length, publication year, and readability and jargon scores of the original funding proposal.

**Results:**

Readability scores ranged from 1.1 to 70.8, with an average FRE score of 39.0 (95% CI 38.4-39.7). Moreover, 2.8% (35/1241) of the PLSs had an FRE score classified as “plain English” or better; none had readability scores in line with the average reading age of the UK population. Jargon scores ranged from 76.4 to 99.3, with an average score of 91.7 (95% CI 91.5-91.9) and 21.7% (269/1241) of the PLSs had a jargon score suitable for general comprehension. Variables such as research topic, funding program, and project size significantly influenced readability and jargon scores. The biggest differences related to the original proposals: proposals with a PLS in their application that were in the 20% most readable were almost 3 times more likely to have a more readable final PLS (incidence rate ratio 2.88, 95% CI 1.86-4.45). Those with the 20% least jargon in the original application were more than 10 times as likely to have low levels of jargon in the final PLS (incidence rate ratio 13.87, 95% CI 5.17-37.2). There was no observable trend over time.

**Conclusions:**

Most of the PLSs published in the NIHR’s National Journals Library have poor readability due to their complexity and use of jargon. None were readable at a level in keeping with the average reading age of the UK population. There were significant variations in readability and jargon scores depending on the research topic, funding program, and other factors. Notably, the readability of the original funding proposal seemed to significantly impact the final report’s readability. Ways of improving the accessibility of PLSs are needed, as is greater clarity over who and what they are for.

## Introduction

In recent years, the idea that science should involve and be accessible to nonscientists has grown. Activities such as patient and public involvement, citizen science, open science, and research coproduction represent different facets of this development and are grounded in both practical and normative motives [1-7]. In health research, one aspect of this involves writing a plain language summary (PLS), sometimes also called a “plain English summary” or lay summary. PLSs are short summaries of a study or project intended to increase its accessibility to nonspecialists. Many regulatory agencies and research funders now require them. For example, the European Union requires a PLS as part of the reporting of all clinical trials [8], and PLSs must be included in all systematic reviews published in the Cochrane Library [9] and all proposals submitted to funders such as the Medical Research Council and the National Institute for Health and Care Research (NIHR), the 2 major state-backed funders of health research in the United Kingdom.

There is evidence to suggest that PLSs do not improve accessibility to the extent we might hope. For example, patients enrolled in clinical trials often have a partial or incorrect understanding of the trial in which they are participating, such as its risks and benefits, despite requirements that they be informed about these issues [10-12]. Pharmaceutical and other industry groups have proposed standards for the preparation of PLS [13-15]. Existing guidelines on how to write a PLS vary and are sometimes contradictory [16] but often highlight issues around readability and avoidance of jargon [17]. Studies of how research findings are disseminated to nonspecialists usually focus on potential users of research and what they need to do to understand research better rather than on examining how characteristics of researchers and research settings influence dissemination to nonacademic audiences [18,19]. Like those who develop public health guidelines, researchers may emphasize internal validity (confidence in the reliability of the results) over external validity (whether and how the results can be applied in other places) [20]. Studies of public health researchers [21] and dissemination and implementation researchers [22,23] found that the context in which research is funded and conducted influences efforts to communicate it to nonspecialists, but we are aware of no studies comparing across fields or subfields. More knowledge about how different characteristics of research influence attempts to communicate the research could help improve communication in the future.

Our aim was to assess the readability and level of jargon in the PLSs of research funded by the United Kingdom’s NIHR. The NIHR is the largest single funding body in the United Kingdom and the largest national clinical research funder in Europe [24]. Full reports of all projects funded in its major research programs are published on the web. Since 2014, it has been obligatory for these to include a PLS that sets out a clear, simple summary of research in a way that is accessible to nonspecialists and members of the public. NIHR guidance for researchers on PLSs [25] is that they should follow “a few simple rules” that include “avoid, wherever possible, jargon, abbreviations, and technical terms,” “avoid complicated language or uncommon words,” and “keep sentences short.” In this study, we addressed 3 questions about these PLSs: How readable are they? How much jargon do they contain? Are readability and use of jargon influenced by study characteristics such as topic and size?

## Methods

### Overview

Our data came from all NIHR Journals Library reports published from mid-2014, when the requirement for inclusion of a PLS was introduced, to mid-2022 (May 30). We downloaded the full text of each report from the NIHR website, where they are publicly available [26]. We then used a purpose-written computer program (written in the computing language called Python) to go through each text and find the PLSs. In a few instances, we could not process the reports in this way, in which case we looked up the PLS manually. We had complete data on 1241 PLSs that were part of reports published in the NIHR Journals Library. Apart from 5 reports that did not have a PLS, we included all the reports published during this period.

### Ethical Considerations

Our study was based on open-access data related to academic publications and we did not engage directly with the individuals or groups making or receiving these publications. As such, our study was exempt from ethical review as per the terms of the authors’ institutional ethics policy and framework (University of Exeter Research Ethics Policy and Framework, Paragraph 4.3.1).

### Outcome Variables

We measured readability using the Flesch Reading Ease (FRE) formula. The FRE is often used in analyzing written health information [27] as well as other scientific texts [28,29] and is based on the idea that longer words and longer sentences make a text less readable. Each text is given a score that gets lower in proportion to the number of longer words and sentences used, so a higher score indicates a text that is easier to read. The formula is as follows:



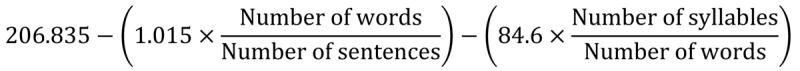



FRE scores can be categorized as “extremely easy,” “very easy,” “fairly easy,” and so on, down to “very difficult,” as well as by approximate reading age (Table 1). We used a short prewritten computer program [30] to calculate readability scores for each PLS. In the rest of this document, when we refer to readability scores, we mean FRE scores calculated in this way.

**Table 1:** Distribution of summaries by Flesch Reading Ease score classification.

			Approximate reading age (years)		Difficulty		Values, n (%)
**Readability score**							
	≥100	Up to 10		Extremely easy		0	
	90-100	11		Very easy		0	
	80-90	12		Easy		0	
	70-80	13		Fairly easy		3 (0.2)	
	60-70	14-15		Plain English		32 (2.6)	
	50-60	16-18		Fairly difficult		169 (13.6)	
	30-50	Undergraduate: 18-21		Difficult		768 (61.9)	
	0-30	Postgraduate: ≥21		Very difficult		269 (21.7)	

We measured jargon using a calculator called the “De-Jargonizer.” It was created to help scientists engage with the public, and it identifies jargon based on the frequency with which words appear in everyday English usage. The developers of the calculator analyzed more than 90 million words used in around 250,000 papers on the British Broadcasting Corporation websites (including news, sports, and science pages) [31] during the years 2012-2015. Based on existing work about how commonly words are used and understood in everyday communication, they categorized the words into high frequency (belonging to the 2000 most common word families, which each appeared more than 1000 times), mid-frequency (appearing between 50 and 1000 times), and jargon (fewer than 50 appearances). Acronyms, which can often be part of jargon, are treated the same way as words, which means that common acronyms such as NHS (National Health Service) or USA (United States of America) fall into the “high frequency” category. Full details of how the calculator was put together, including testing and validation, have been published [32], and a web-based version of the calculator contains a description and additional details [33].

The developers of the calculator also created a score to indicate how suitable a text was for a general audience. If a text uses only common words, the score is 100; lower scores indicate more use of mid-frequency and jargon words. The score is calculated using this formula:



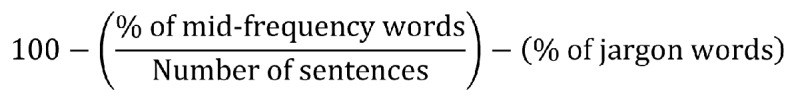



We downloaded the source code [34] for the calculator and used it to work out a jargon score for each PLS. A higher score means that less jargon was used.

We created additional outcome variables to identify PLSs that were better in terms of readability and jargon. For readability scores, we focused on summaries with scores of >50: those classed as “fairly difficult to read” or better. A total of 204 summaries (16.4%, 204/1241) fell into this category. The starting point of this study related to the average reading age in the United Kingdom (see the “Patient and Public Involvement” section). We created an outcome variable showing whether a readability score was suitable for a reading age of 9 years—that is, a readability score of 100 or above (Table 1). However, no summaries fell into this category. Rakedzon and colleagues [32] refer to 2 levels of jargon (2% or 5%) as “recommended for general comprehension.” We used the more generous 5% score and categorized scores of more than 95 as having low levels of jargon. A total of 269 (21.7%, 269/1241) summaries fell into this category. Multimedia Appendix 1 shows examples of PLSs with high and low readability and jargon.

### Study Characteristics

We used the following information relating to each PLS in our analyses:

1. Research topic: NIHR classifies all research projects using the UK Clinical Research Collaboration Health Research Classification System. This is a system for categorizing health research funding and is used to allow funders and others to assess funding schemes [35]. We used the Health Categories dimension of the Health Research Classification System, which groups each project into 1 or more of 21 categories. In our analysis, we included categories if they had at least 50 projects associated with them, which left us with 12 categories: cancer and neoplasms, cardiovascular, infection, mental health, metabolic and endocrine, musculoskeletal, neurological, oral and gastrointestinal, reproductive health and childbirth, respiratory, stroke, and “generic health relevance.”
2. Funding program: Reports published in the National Journals Library relate to 5 NIHR funding programs: Efficacy and Mechanism Evaluation, Health Technology Assessment, Health and Social Care Delivery Research, Programme Grants for Applied Research, and Public Health Research. Of these, the Efficacy and Mechanism Evaluation program is generally considered most “upstream” (closer to basic than applied science), and the Health and Social Care Delivery and Public Health Research programs are most “downstream” (most applied). Details of all NIHR programs are available on the web [36].
3. Project size: We wanted to know whether the size of a project made a difference to the PLS, and we used the amount of funding as an approximate measure for this. Smaller projects tend to be more focused and contained; larger projects may have more resources to move around and support activities such as public engagement (which does not necessarily mean that a “better” PLS will be produced). We categorized projects by size by ranking them in terms of the amount of funding received and then sorting them into 5 equal groups: top 20%, next 20%, and so on.
4. Length in words: When preparing their report for the National Journals Library, authors are asked to write a PLS of up to 300 words, but some write shorter summaries, and some write longer ones. Studies of patient-information leaflets in trials have found that long [37,38] and short [39] texts each have problems associated with them regarding readability and clarity, and we wanted to see whether the length of the PLS made a difference.
5. Readability and jargon scores of the original funding proposal: Information about all projects funded by the NIHR is publicly available on the web, including a copy of the PLS submitted as part of the original funding proposal. The difference between this and the final National Journals Library PLS is that the original one sets out what the researchers proposed to do; the PLS in the National Journals Library summarizes what they ultimately did and found. NIHR instructs writers of summaries to “follow the same principles and procedures as in writing the plain language summary that accompanied your funding submission” [40], and we wanted to find out whether original and final report summaries were written in similar ways. Just as we did for the National Journals Library PLSs, we calculated readability and jargon scores for each of the funding proposal PLSs. We categorized these scores by ranking them and then sorting them into 5 equal groups.
6. Publication year: PLSs became a requirement in mid-2014, and we downloaded our data in mid-2022 (July 22). We wanted to see whether the readability and use of jargon in PLSs had changed over time and did this by categorizing each one according to the year it was published.

### Analysis

We analyzed our data using Stata/SE (version 17.0; StataCorp LLC) and Microsoft Excel (version 2409; Microsoft Corporation). For each of the study characteristics described in the preceding section (research topic, funding program, project size, length in words, readability or jargon used in the original proposal, and publication year), we calculated descriptive statistics on the number of PLSs in each category or, where categories were of equal size, the range covered by the category. For each category, we estimated the average readability and jargon score, the percentage of summaries with readability scores higher than 50, the percentage of summaries with jargon scores higher than 95, and the 95% CIs for these estimates. We looked at the relationship between readability and jargon scores by calculating their pairwise (Pearson) correlation. We estimated incidence rate ratios and 95% CIs of PLSs in the hardest-to-read categories (readability scores of >50 and jargon scores of >95) using a generalized linear model with a modified Poisson approach and robust error variances [41]. We report the results of a model in which all study characteristics were entered simultaneously.

### Patient and Public Involvement

This study was prompted by the assertion made at a patient and public involvement meeting, attended by AK (who is a patient and member of the public but not a researcher) and hosted by a leading UK research-funding charity, that the average reading age of the UK population is 9 years. This assertion (along with a few variations) can be found on numerous websites by searching on the web for the term “UK average reading age” (we subsequently found that these figures come from the UK Government’s Skills for Life Survey [42]). This raised the question: if this is the case, how accessible to the general population are the PLSs routinely produced in health research funding applications and reports? AK and IAL’s discussions about how to address this question led to the writing of this paper. AK has been involved throughout and is a coauthor.

## Results

Readability scores in our sample ranged from 1.1 to 70.8. The mean (average) FRE score was 39.0 (95% CI 38.4-39.7), and the median (middle) score was 39.8. The distribution of scores across readability categories is shown in Table 1. Around one-fifth of summaries had a score below 30, “very difficult to read.”

Jargon scores in our sample ranged from 76.4 to 99.3. The mean (average) jargon score was 91.7 (95% CI 91.5-91.9), and the median (middle) score was 92.4. The distribution of scores across jargon categories is shown in Table 2. Around one-fifth of summaries had a score of 95 or above, suggesting that they would be suitable for a general audience.

**Table 2:** Distribution of summaries by jargon score.

Jargon score	Values, n (%)
95-100 (least jargon)	269 (21.7)
90-95	592 (47.7)
85-90	300 (24.2)
85 or lower (most jargon)	80 (5.4)

The pairwise correlation between readability scores and jargon scores was 0.249 (*P*<.001), which suggests that when one score is higher, the other is also likely to be higher but that the relationship is moderate. Sixty-six summaries (5.3%, 66/1241) were in both the “easier to read” and “least jargon” categories.

Table 3 shows, for each of the study characteristics, the number of summaries in each category (where relevant) and the mean readability and jargon scores for that category. We found statistically significant variations in estimated readability and jargon scores between categories for each study characteristic.

**Table 3:** Distribution of summaries, mean readability scores, and mean jargon scores in relation to each study characteristic.

			Number in each category, n (%)		Mean readability score (higher=easier to read; scores of ≥60 are “plain English,” 50-60 are suitable for people educated to high-school level, 30-50 for undergraduate level, and 30 and below for postgraduate level) (95% CIs)		Mean jargon score (higher=less jargon; scores of ≥95 are suitable for a general audience) (95% CIs)
**Research topic^a^**							
	Cancer and neoplasms	164 (13.2)		39.9 (37.9-41.9)		91.2 (90.6-91.9)	
	Cardiovascular	165 (13.3)		41.6 (39.8-43.4)		91.6 (91.0-92.1)	
	Generic health relevance	271 (21.8)		37.7 (36.3-39.1)		94.2 (93.9-94.4)	
	Infection	93 (7.5)		38.4 (36.2-40.6)		90.3 (89.5-91.1)	
	Mental health	211 (17.0)		38.2 (36.7-39.7)		93.1 (92.7-93.5)	
	Metabolic and endocrine	78 (6.3)		42.6 (39.9-45.2)		92.4 (91.6-93.2)	
	Musculoskeletal	57 (4.6)		37.8 (33.3-40.3)		91.2 (90.2-92.2)	
	Neurological	81 (6.5)		38.0 (35.3-40.7)		92.4 (91.6-93.2)	
	Oral and gastrointestinal	106 (8.5)		42.1 (39.9-44.2)		91.7 (91.0-92.5)	
	Reproductive health and childbirth	96 (7.7)		41.6 (39.6-43.7)		91.2 (90.5-91.9)	
	Respiratory	56 (4.4)		41.3 (38.2-44.4)		91.0 (90.1-92.0)	
	Stroke	110 (8.9)		41.7 (39.5-43.9)		92.9 (92.3-93.5)	
**Funding program^b^**							
	Efficacy and Mechanism Evaluation	81 (6.5)		38.8 (36.4-41.2)		88.4 (87.7-89.1)	
	Health Technology Assessment	630 (50.8)		39.4 (38.5-40.3)		90.3 (90.0-90.6)	
	Health and Social Care Delivery Research	327 (26.4)		37.3 (36.1-38.6)		94.2 (93.9-94.5)	
	Programme Grants for Applied Research	96 (7.7)		37.5 (35.3-39.6)		92.8 (92.2-93.4)	
	Public Health Research	107 (8.6)		43.4 (41.3-45.6)		94.0 (93.6-94.5)	
**Publication year^b^**							
	2014	91 (7.3)		37.6 (35.4-39.8)		92.5 (91.8-93.2)	
	2015	170 (13.7)		38.7 (36.8-40.6)		91.5 (90.9-92.0)	
	2016	168 (13.5)		37.5 (35.5-39.5)		91.2 (90.5-91.9)	
	2017	147 (11.8)		39.2 (37.6-40.9)		91.8 (91.2-92.4)	
	2018	142 (11.4)		41.1 (39.4-42.8)		91.9 (91.4-92.5)	
	2019	153 (12.3)		41.3 (39.6-43.0)		91.7 (91.0-92.3)	
	2020	154 (12.4)		38.7 (36.7-40.7)		92.1 (91.4-92.7)	
	2021	145 (11.6)		38.7 (36.8-40.5)		91.7 (91.0-92.4)	
	2022	71 (5.7)		37.1 (34.2-39.9)		91.5 (90.6-92.5)	
**Project size**							
	Smallest 20%			36.9 (35.5-38.4)		91.4 (90.9-91.9)	
	2			38.1 (36.6-39.7)		92.7 (92.2-93.1)	
	3			39.5 (38.1-40.9)		93.1 (92.7-93.6)	
	4			40.2 (38.8-41.6)		90.5 (89.9-91.0)	
	Largest 20%			40.3 (38.9-41.7)		91.0 (90.5-91.4)	
**Length of summary**							
	Shortest 20%			38.6 (37.0-40.1)		91.6 (91.1-92.2)	
	2			39.2 (37.7-40.7)		92 (91.5-92.4)	
	3			39.3 (37.9-40.6)		91.2 (90.7-91.7)	
	4			40.9 (39.5-42.2)		92.8 (92.3-93.2)	
	Longest 20%			37.2 (35.7-38.6)		91.1 (90.5-91.6)	
**Scores in original proposal^c^**							
	Lowest 20% of original scores			35.3 (33.8-36.8)		88.0 (87.5-88.4)	
	2			36.8 (35.3-38.4)		90.5 (90.1-91.0)	
	3			39.4 (38.0-40.7)		92.2 (91.8-92.6)	
	4			39.7 (38.4-40.9)		93.3 (92.9-93.6)	
	Highest 20% of original scores			43.9 (42.5-45.3)		94.7 (94.4-94.9)	

^a^Some summaries were associated with more than 1 area of research, so the total percentage does not add up to 100.

^b^Percentages may not add up to 100 because of rounding.

^c^Readability scores are categorized by the readability score of the original funding proposal; jargon scores are categorized by the jargon score of the original funding proposal.

For the research topic, the highest estimated readability score (most readable) was in the “Metabolic and endocrine” category, and the lowest score (least readable) was for “Generic health relevance.” However, the “Generic health relevance” category was associated with the highest estimated jargon score (least jargon), and the lowest score (most jargon) was for “Infection.”

For the funding program, the highest estimated readability score was associated with the Public Health Research program, and the lowest was in Health and Social Care Delivery Research. In contrast, the Health and Social Care Delivery Research had the highest estimated jargon score (least jargon). The lowest jargon score (most jargon) was associated with the Efficacy and Mechanism Evaluation program.

For project size, mean readability scores but not jargon scores rose as projects got larger. The highest estimated readability score was associated with the largest projects (top 20% of funding), and the lowest readability was associated with the smallest (bottom 20%). There was no clear pattern of variation in jargon scores.

For length of summary, longer summaries were associated with better readability and less jargon, but only up to a point. Scores appeared to rise across the first 80% of summaries when ranked by length but then dipped so that the lowest scores were in the longest 20% of summaries.

For publication year, the lowest readability score was for 2022, but data for that year were incomplete at the time we collected our data. Readability and jargon scores varied by year, but there did not appear to be any trend in either score over time.

Our estimates of scores in summaries in relation to scores in original proposals rose steadily across the categories from lowest to highest proposal scores for both readability and jargon. For both readability and jargon, the highest estimated scores were for PLSs that had the highest scores in the proposals, and the lowest estimated scores were for PLSs that had the lowest scores in the proposals.

**Figure 1:**
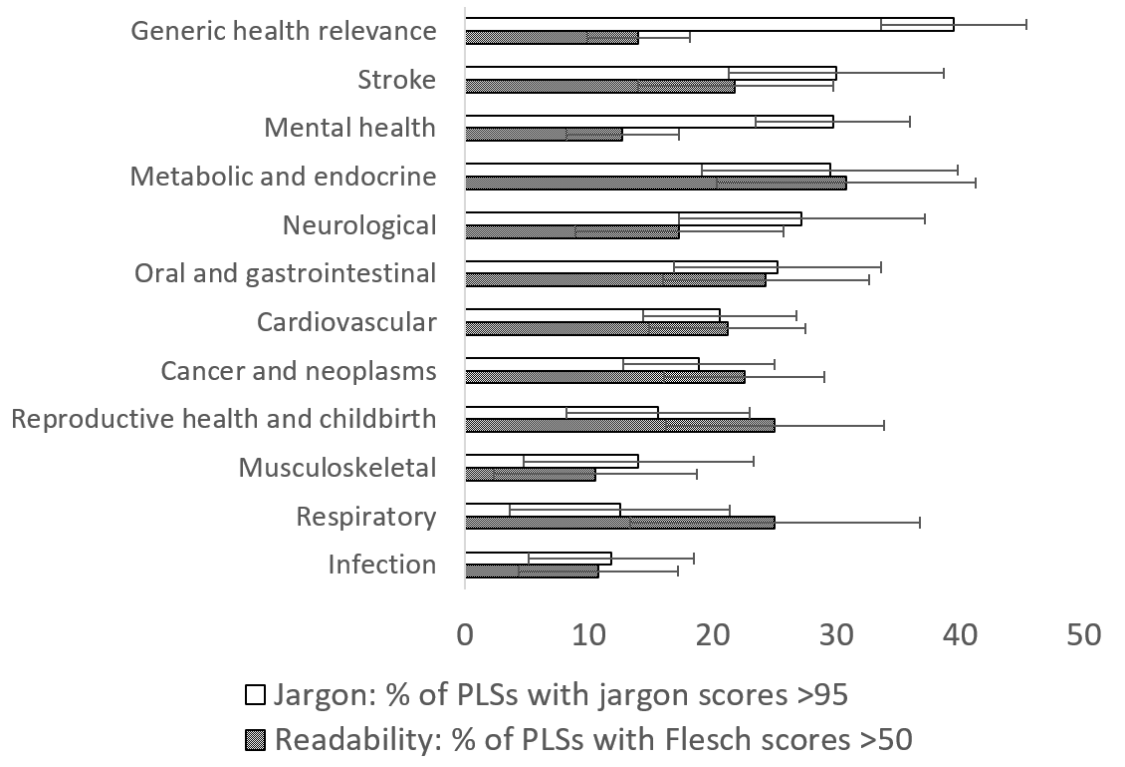
Percentage of summaries with readability scores >50 (“fairly difficult to read” or better) and with jargon scores >95 (low level of jargon), by research topic, with 95% CIs. PLSs: plain language summaries.

**Figure 2:**
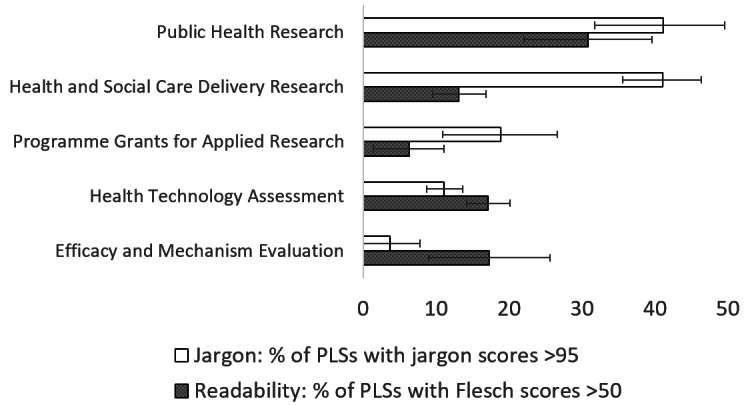
Percentage of summaries with readability scores >50 (“fairly difficult to read” or better) and with jargon scores >95 (low level of jargon), by National Institute for Health and Care Research funding program, with 95% CIs. PLSs: plain language summaries.

**Figure 3:**
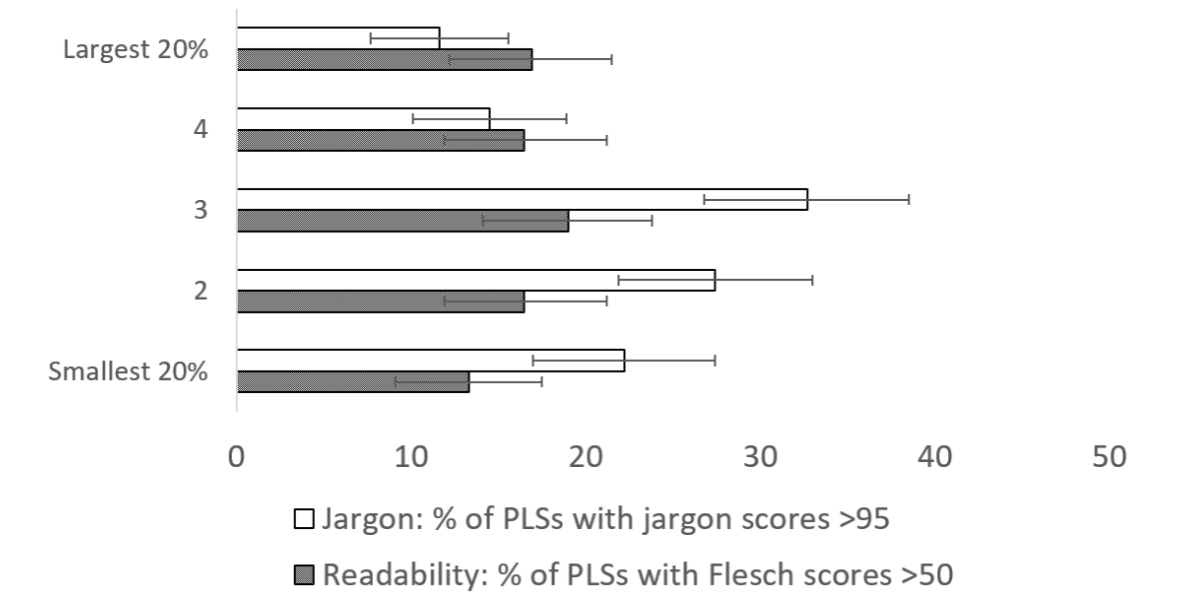
Percentage of summaries with readability scores >50 (“fairly difficult to read” or better) and with jargon scores >95 (low level of jargon), by project size, with 95% CIs. PLSs: plain language summaries.

**Figure 4:**
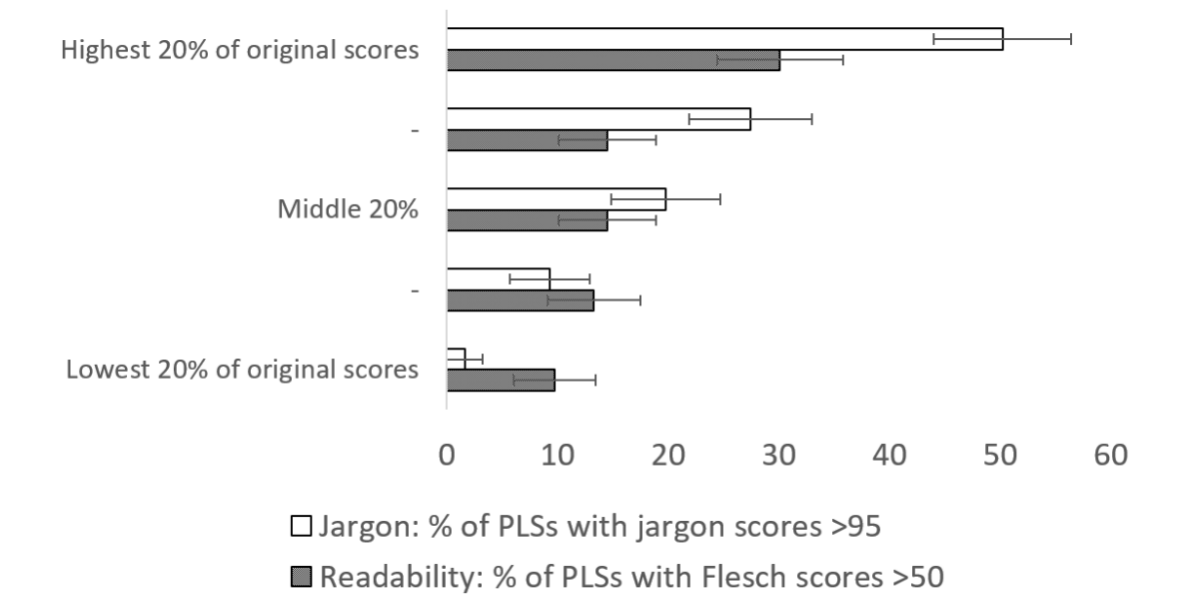
Percentage of summaries with readability scores >50 (“fairly difficult to read” or better) and with jargon scores >95 (low level of jargon), by readability scores or jargon scores (as appropriate: readability scores are categorized in relation to the readability score of the original funding proposal, and jargon scores are categorized in relation to the corresponding original jargon score) in original proposals, with 95% CIs. PLSs: plain language summaries.

The proportion of summaries that have readability scores over 50 (“fairly difficult to read” or better) and jargon scores >95 (low level of jargon) are shown in Figure 1 (by research topic), Figure 2 (by funding program), Figure 3 (by project size), and Figure 4 (by scores in original proposals).

Finally, Table 4 shows the incidence rate ratios of having each of these “better” scores when all study characteristics are included in the same model. This is useful because it allows us to assess whether the differences we have observed are still there when the other differences between summaries are accounted for. It could be, for example, that summaries in the Public Health Research program were more likely to be in the easier-to-read category, compared with Programme Grants for Applied Research, because of differences in project size or research topic. The results of the regression suggest, however, that even when other differences (eg, size, topic, year) are controlled for, Public Health Research summaries are approximately 5 times as likely as Programme Grant summaries to be more readable. Similarly, we can say that projects for which the original proposal had little jargon are markedly more than 10 times as likely to have little jargon in their final reports when compared with those with lots of jargon in the original proposal, even when differences in size, topic, and so on are accounted for.

**Table 4:** Incidence rate ratios and 95% CIs from regression analyses including all study characteristics for each outcome of interest: readability scores >50 (“fairly difficult to read” or better) and jargon scores >95 (low level of jargon).

			Readability scores ≥50 (“fairly difficult to read” or better), incidence rate ratio (95% CI)		Jargon scores ≥95 (suitable for a general audience), incidence rate ratio (95% CI)
**Research topic**					
	Cancer and neoplasms	1.12 (0.70-1.79)		0.84 (0.45-1.56)	
	Cardiovascular	0.95 (0.57-1.58)		0.99 (0.55-1.78)	
	Generic health relevance	0.90 (0.57-1.41)		1.86 (1.32-2.60)	
	Infection	0.55 (0.31-0.97)		1.25 (0.75-2.09)	
	Mental health	0.73 (0.47-1.14)		1.71 (1.26-2.32)	
	Metabolic and endocrine	1.72 (1.08-2.76)		1.26 (0.80-1.98)	
	Musculoskeletal	0.68 (0.31-1.49)		1.17 (0.63-2.15)	
	Neurological	1.05 (0.63-1.75)		1.74 (1.21-2.50)	
	Oral and gastrointestinal	1.10 (0.63-1.93)		1.10 (0.66-1.86)	
	Reproductive health and childbirth	1.45 (0.95-2.20)		1.32 (0.84-2.09)	
	Respiratory	1.72 (1.03-2.87)		0.90 (0.44-1.86)	
	Stroke	0.50 (0.23-1.09)		1.69 (0.98-2.92)	
**Funding Program**					
	Efficacy and Mechanism Evaluation	2.59 (1.01-6.61)		1	
	Health Technology Assessment	3.00 (1.31-6.87)		2.25 (0.74-6.85)	
	Health and Social Care Delivery Research	2.56 (1.05-6.25)		4.08 (1.33-12.54)	
	Programme Grants for Applied Research	1		3.27 (1.00-10.66)	
	Public Health Research	5.35 (2.13-13.4)		3.87 (1.25-11.96)	
**Publication year**					
	2014	0.78 (0.4-1.54)		0.62 (0.38-1.00)	
	2015	0.95 (0.56-1.62)		0.72 (0.47-1.11)	
	2016	1.00 (0.60-1.67)		0.89 (0.58-1.37)	
	2017	0.82 (0.47-1.44)		1.05 (0.70-1.58)	
	2018	1.01 (0.64-1.61)		0.81 (0.54-1.22)	
	2019	1		1	
	2020	1.25 (0.82-1.89)		1.22 (0.88-1.70)	
	2021	0.82 (0.50-1.32)		1.49 (1.05-2.12)	
	2022	0.65 (0.34-1.25)		1.03 (0.67-1.60)	
**Project size**					
	Smallest 20%	1		1	
	2	1.19 (0.77-1.83)		0.89 (0.67-1.20)	
	3	1.36 (0.89-2.06)		1.01 (0.76-1.35)	
	4	1.18 (0.76-1.83)		0.69 (0.49-0.99)	
	Largest 20%	1.64 (1.06-2.55)		0.59 (0.37-0.97)	
**Length of summary**					
	Shortest 20%	1		1	
	2	1.07 (0.71-1.61)		1.09 (0.81-1.47)	
	3	0.81 (0.51-1.27)		0.67 (0.47-0.97)	
	4	1.07 (0.67-1.69)		0.93 (0.65-1.32)	
	Longest 20%	0.69 (0.42-1.13)		0.74 (0.51-1.10)	
**Scores in original proposal^a^**					
	Lowest 20% of original scores	1		1	
	2	1.39 (0.85-2.26)		4.53 (1.62-12.68)	
	3	1.40 (0.87-2.26)		7.26 (2.66-19.79)	
	4	1.48 (0.91-2.40)		8.74 (3.23-23.63)	
	Highest 20% of original scores	2.88 (1.86-4.45)		13.87 (5.17-37.2)	

^a^We analyzed readability score outcomes in relation to the readability score of the original funding proposal and jargon score outcomes in relation to the jargon score of the original funding proposal.

## Discussion

### Main Findings

Our findings suggest that the PLSs published in the NIHR’s Journals Library are often difficult to read and likely inaccessible to a general audience. Despite the NIHR’s advice to avoid jargon and complicated words and to keep sentences short, many published summaries had lots of jargon and poor readability. We analyzed more than 1200 summaries and found none with readability scores suggesting that they would be accessible to people with the average UK reading age of 9 years.

Readability and jargon scores varied significantly in relation to research topic (where “Metabolic and endocrine” projects did best), funding program (where projects in the Public Health Research Programme did best), and, most noticeably, in relation to how readable the original funding proposal was. The relationship between original funding proposal summaries and final report summaries is notable because it suggests that some authors are consistently better (or worse) at writing accessible summaries.

Readability scores and jargon scores were correlated but did not always coincide. For instance, summaries in the “Generic health relevance” category used relatively few jargon words but were not very readable. The opposite was true of larger projects, which, compared with small projects, had more readable summaries and less jargon. The Efficacy and Mechanism Evaluation funding program was also associated with summaries that were moderately readable yet had high levels of jargon. These differences suggest that summaries may be accessible or easy to read in some ways but not in others and that relying on a single measure to assess a text may miss important aspects of readability. We found no pattern over time: looked at year-on-year, there are differences but no upward or downward trend in either readability or jargon scores.

### Comparisons With Other Studies

The mean FRE score we found was higher than the mean score of 23.6 reported for PLSs in 2 psychology journals [43] and the mean of 21 found in a sample taken from the Physiotherapy Evidence Database [44]. A study of research on cystic fibrosis found that the FRE score of PLSs in journals was 43.3 and in Cochrane reviews was 46.3 [45]. An analysis of medicine information sheets produced by the professional associations of rheumatologists in 3 countries found that the average FRE score for those from Australia was 50.8, from the United Kingdom was 48.5, and from Canada was 66.1 [46]; in patient information leaflets produced by the British Association of Dermatologists, the mean score was 52.2 [47]. An analysis of Cochrane PLSs, which used a different readability formula, concluded that most would be difficult to read for someone with no medical education [48] and is in line with a previous analysis of Cochrane PLSs that found that they were very heterogeneous and often failed to adhere to standards [49]. Comparing scores across different samples of texts is not straightforward because of differences in the intended audiences for each. What is clear is that the level of readability we found is markedly worse than that recommended for texts in plain language and suitable for a general audience, which is a score of 60 or above (Table 1).

We are aware of only 1 previous use of a jargon calculator to assess PLSs by the developers of the calculator we used [32]. They compared levels of jargon in academic abstracts and lay summaries in 2 journals, *PLOS Computational Biology* and *PLOS Genetics*. Although they found that the PLSs contained less jargon than the abstracts, they—like us—found that jargon use in the summaries was significantly higher than the recommended levels. We used a cutoff of 5% to identify summaries with low levels of jargon and identified that only 21.7% (269/1241) of papers met this criterion; of the summaries in our sample, only 1.0% (13/1241) met the more stringent 2% cutoff proposed by Rakedzon and colleagues [32].

### Strengths and Weaknesses of This Study

Previous studies of PLSs have focused on specific research areas (such as physiotherapy) or methods (such as reviews). Our broader approach has enabled us to address a part of what Uphold and colleagues [18] described as a critical gap in the dissemination and implementation literature, the analysis of how researcher characteristics and environmental determinants influence attempts to disseminate research findings. We also looked at both readability and jargon, whereas previous studies have focused on one or the other.

FRE scores are widely used [27], are recommended by NIHR and other funders as a way of assessing the readability of PLSs [25], and—as we have used them here—provide a way of looking at large numbers of texts and identifying trends and tendencies in readability. We recognize, all the same, that readability indices are an imperfect way of assessing texts. They can be misleading and have little to do with how easy a text is to understand [50-52], and scores may be inconsistent when tested across different pieces of software because of formatting or other differences [27]. Readability indices also capture only 1 aspect of how science is communicated to the public. There are more sophisticated ways of understanding [53,54], conducting [55,56], and assessing [57-59] science communication. Our approach focused on summaries written by research teams and could be extended by looking at the perceptions and responses of readers and evaluating the impact of summaries [60-62]. We had no information on the extent to which teams responded to NIHR’s “strong encouragement” to involve a nonacademic member of the public in writing the PLS [25], so we cannot comment on whether this alters their content and presentation.

The way we have assessed jargon focuses on single words and ignores the use of phrases that might otherwise count as jargon. Multimedia Appendix 1 contains some examples of this. For example, “confidence” and “interval” are both classed as mid-frequency words in our jargon calculator, but the phrase “confidence interval,” often used in reporting statistical estimates in health studies (as in this paper), would probably be considered jargon. The same applies to longer jargon terms such as “incremental cost-effectiveness ratio,” which has a specific technical meaning but which the jargon calculator does not pick up on. We have not accounted for other aspects of summaries that can affect how readable they are, such as the use of numbers and statistics.

The summaries we looked at were all funded by a single large funder (NIHR) and relate to research done in a single country (the United Kingdom). Over the past 3 decades, the United Kingdom has more swiftly embedded public involvement and engagement in health research than most other countries [63,64]. While we may appear critical of NIHR, this study has been possible only because NIHR requires researchers to write summaries and then makes these publicly accessible. NIHR sponsors the UK Standards for Public Involvement, intended as a “description of what good public involvement looks like,” and 1 of the 6 standards is “Communication—use plain language for well-timed and relevant communications, as part of involvement plans and activities” [65]. The NIHR also says that it is “the world’s ﬁrst health research funder to publish comprehensive accounts of its funded research within its own publicly and permanently available journals” [26].

The PLSs included in National Journals Library publications are central to these 2 commitments—to make publicly available details of the work it funds and to communicate in plain language—and are “in keeping with the NIHR Journals Library’s commitment to accessibility” [26]. They also relate to a substantial investment: the cost of the research represented in the reports at which we looked is not easy to calculate, but NIHR expenditure on these funding streams in financial year 2021/22 was £206.3 million (approximately €241 million/US $268 million), so it seems reasonable to assume that the cost over the years covered here exceeded £1 billion (approximately €1.17 billion/US $1.30 billion). We might expect to see in the work of the NIHR a well-developed set of processes and mechanisms by which to communicate the results of research to the public, and for this reason, we consider the NIHR’s flagship publication stream, the Journals Library, to be an apt focus for our enquiry here.

The extent to which the assessment of readability can be regarded as an indication of comprehension is not included in this study, as there is no existing measure that captures this relationship. Assessing comprehensibility usually involves asking people to read 1 or more texts and then measuring their understanding (eg, as Koops et al [66] did), which would be challenging to do with a large sample of texts such as those in the NIHR Journals Library. In the absence of measurable comprehensibility, assumptions on the accessibility of PLSs deserve scrutiny and a willingness to reconsider the methods of their production.

### Unanswered Questions and Future Research

We would like this study to contribute to a debate on 2 connected questions: What and who are PLSs for?

From a funder’s perspective, PLSs are a relatively cheap and easy way of disseminating research findings. The teams doing the research are responsible for producing a PLS, and this has strengths (the information “straight from the horse’s (or scientist’s) mouth” [67] is presumably less likely to be incorrect or imprecise) as well as weaknesses (researchers often lack training in science communication). Scientific writing can be exclusive: “The language of science, though forward-looking in its origins, has become increasingly anti-democratic… [it] sets apart those who understand it and shields them from those [who] do not” [68] but fluency in this language is necessary for scientists to do their work and have it accepted by other scientists [69]. PLSs could be an important aspect of a more “engaged” university sector [64], but the intended role and audience for PLSs are unclear, and this limits their potential value.

Communicating in a certain way means communicating to a group of people who can understand that type of communication. Attempts to communicate to “the general public” [25] must address multiple audiences [70], but the members of the public being addressed include people who differ widely in their interest, knowledge, and trust in science [71]. Ahmed [72] argues, drawing on the work of Warner [73], that addressing a public generates a public that can be addressed. Saying something in Swahili implies that you are speaking to people who can understand Swahili, and writing something using lots of scientific jargon and complicated sentences implies that you intend it for an audience who can understand such language. The dissemination of research findings has been challenged as a 1-way form of communication that falls short of the principles and expectations of public engagement [74], can be seen as representing public relations rather than science communication [75,76], and contributes to the exclusion of people from low-income, minority ethnic groups [77]. If we regard the production of PLSs as an aspect of Open Science, with its commitments to social engagement in the conduct and outcomes of research [5], we also need to be aware of what is being made visible and invisible in the process [78] and of who is being included and excluded.

Evidence on what is most effective in PLSs of scientific research, in terms of both form and content, is emerging [16,67,79], but there is still much for us to learn about what works, for whom, and why. The value of training to improve science communication is unclear [80], and attempts to improve PLSs have met with mixed results. For example, Kirkpatrick and colleagues [81] tested 2 approaches: having authors rewrite PLSs using new guidance and having an independent medical writer edit the PLS. In each case, a group of nonspecialists rated the revised versions as easier to read but not easier to understand. A service designed to improve recruitment to studies by having trained patients and carers review research documents was successful in reducing the amount of jargon but not in improving readability [82].

Creative approaches to communicating research findings have the potential to enable 2-way communication and flatten hierarchies between scientists and nonscientists [83]. Again, when putting this to the test, results have been mixed: one recent study found that PLSs of published research were more effective than scientific abstracts or graphical abstracts in terms of comprehension and understanding [84], whereas another study concluded that graphical summaries were one of the most preferred formats [85]. A recent review of instructions for authors on writing PLSs found a lot of inconsistency across journals and suggested that consistent instructions could be developed with members of the public [17]. The creation of common standards for summaries has also been proposed as part of the OpenPharma project [13,14]. Expert consensus conference methods have been used to produce recommendations on maximizing the accessibility of study patient-information leaflets and informed-consent forms [86], and a similar approach could potentially be applied to the preparation of PLSs.

Another approach from which we might learn is citizen science. Communication of all aspects of research is fundamental to citizen science, which has been described as one of the most dramatic developments in science communication in decades [87] It emphasizes multidirectional and ongoing communication [88] and recognizes storytelling and visualization as central to this [89]. At least in aspiration, citizen science has the potential to improve and transform science communication at the same time as it empowers and informs citizen scientists [90]. Other approaches to dissemination have emphasized coproduction [91] and community engagement [92]. As Knowles and colleagues noted, “finding mutually acceptable and valuable ways to express findings is yet another area requiring open discussion and negotiation” [93].

### Conclusions

We found that the sample of PLSs that we examined had low readability and contained lots of jargon. Although these things differed in relation to study characteristics, such as topic and size, none of the PLSs had readability scores in line with the average reading age of the UK public. The aims of, and audiences for, these PLSs are unclear, and their place in science communication and public engagement requires further consideration. It is uncertain whether these summaries improve public access to research.

## Data Availability

The data we used came from the texts and metadata of reports published in the NIHR’s Journals Library. This is an open-access resource, and all the data we used are publicly available on the web via the Journals Library website: https://www.journalslibrary.nihr.ac.uk/journals/

## Appendix

Multimedia Appendix 1 Exemplar plain language summaries with high and low readability and high and low jargon scores.

## Abbreviations

FRE: Flesch Reading Ease
NIHR: National Institute for Health and Care Research
PLS: plain language summary
