# Supplementary material for: Jargon and Readability in Plain Language Summaries of Health Research: Cross-Sectional Observational Study
Source: J Med Internet Res. 2025 Jan 13;27:e50862. doi: 10.2196/50862 (PMC11773280; doi:10.2196/50862)
Supplement: Multimedia Appendix 1 [file jmir_v27i1e50862_app1.docx]

## Appendix 1. Examples of Plain Language Summaries with differing combinations of high and low readability and jargon.

In the summaries below, words the jargon calculator categorised as mid-frequency are in *italics* and rare words are underlined.

### High readability (FRE score 70.0), little jargon (jargon score 96.3)

Standing *frames* help people with *cerebral palsy* (CP) to stand when they cannot do this by themselves. They may also be useful for improving body position, *digestion*, bone strength and helping young people to join in with others. However, there is very little scientific evidence (or proof) that they are *helpful*.

Standing *frames* are expensive and can be painful to use. The NHS wants to find out if standing *frames* really work. This study aimed to work out if people would take part in a research trial to find this out. We interviewed young people with CP and asked parents and professionals who work with young people with CP (who use a standing frame) to answer questions in *surveys*. We also asked parents and professionals to take part in focus groups.

We found that lots of things make using a standing frame difficult, such as a lack of time, space or support. Young people want their standing *frames* to help them take part in things. Some parents and professionals, such as physiotherapists, think that it is dangerous to stop using a standing frame for a long time but may stop for a short time for a research trial.

The people who took part in the study believe that standing *frames* help in lots of different ways, although most think that more research is needed.

This study shows that in a future trial the most important things to find out are:

- Does using a standing frame help a young person to feel more satisfied, *healthier* or *happier*?

- Do they make it easier for them to take part in activities?

- Can they help with body *functions* (like *breathing* and *digestion*) and body structure (like bones and *muscles*)?

A research trial needs to be carefully planned to help people to take part.

Goodwin J, Lecouturier J, Basu A, Colver A, Crombie S, Smith J, _et al._ Standing frames for children with cerebral palsy: a mixed-methods feasibility study. Health Technology Assessment 2018;22(50). <https://doi.org/10.3310/hta22500>

### Low readability (FRE score 22.8), little jargon (jargon score 96.7)

Health-care *scandals*, such as the most recent at Mid *Staffordshire* NHS Foundation Trust, have demonstrated that uncaring practices can *flourish* in hospitals when the *organisational* context goes wrong. When this happens, patients not only have *unpleasant* experiences during care but can be hurt or even die as a *consequence*. Hospital boards have ultimate responsibility for *safeguarding* the care provided in their organisation, yet recent high-profile reports on serious *failings* in the quality of hospital care in the NHS have raised concerns over the ability of boards to *discharge* their duties effectively in this area. This research *seeks* to better understand the processes associated with the effective board governance of safe care. Based on a review of the available evidence and research in hospitals we examine in detail what hospital boards actually do in relation to *safeguarding* care in their organisation; for example, how much time they spend discussing patient safety issues, the types of information boards use to assess the quality of care in their organisation, what training and skills board members have in relation to patient safety and how factors, such as external financial *incentives*, influence what boards do in relation to patient safety. We found that what boards do and focus on is related to how their hospital *reacts* to and deals with patient safety issues. In particular, we found a link between self-assessed competencies of boards and whether or not its staff felt able to *openly* report patient safety-related problems and incidents.

Mannion R, Freeman T, Millar R, Davies H. Effective board governance of safe care: a (theoretically underpinned) cross-sectioned examination of the breadth and depth of relationships through national quantitative surveys and in-depth qualitative case studies. Health Services Delivery Research 2016;4(4). <https://doi.org/10.3310/hsdr04040>

### High readability (FRE score 54.1), lots of jargon (jargon score 86.8)

In patients with cancer, *fluid* can build up in the space between the chest wall and *lung*, causing breathlessness. The *fluid* can be *drained* using a small tube *inserted* between the *ribs* under local *anaesthetic*. However, it often recurs. To avoid this, doctors usually *inject* talc *powder* (mixed into a slurry) back down the *drainage* tube to try to ‘stick’ the *lung* to the inside of the chest wall. If successful, this *prevents* the *fluid reforming*. This procedure is called pleurodesis.

An alternative is to *insert* a camera into the chest under light sedation and local *anaesthetic* (a ‘thoracoscopy’) and *spray* talc directly onto the inside of the chest wall (poudrage). This may be more effective, although this has not been proven and it is a slightly more complex procedure.

Therefore, this trial was conducted to see if poudrage was more effective than slurry. A total of 330 patients were *recruited* from 17 UK hospitals who had chest *fluid* due to cancer. They were divided *evenly*, with half receiving standard *drainage* and slurry and the other half receiving a thoracoscopy and poudrage. They were followed up for 6 months. We measured how many experienced a *recurrence* in *fluid* build-up 3 months after treatment, as well as other *impacts*, including if there was any difference in the long-term costs.

No difference in clinical *effectiveness* was found between talc poudrage and talc slurry. Poudrage was unlikely to be cost-effective.

In *summary*, the researchers *conclude* that slurry is likely to be the *preferable* method.

Bhatnagar R, Luengo-Fernandez R, Kahan BC, Rahman NM, Miller RF & Maskell NA. Thoracoscopy and talc poudrage compared with intercostal drainage and talc slurry infusion to manage malignant pleural effusion: the TAPPS RCT. Health Technol Assess 2020;24(26). <https://www.journalslibrary.nihr.ac.uk/hta/hta24260#/abstract>

### Low readability (FRE score 6.6), lots of jargon (jargon score 78.0)

Thrombocytopenia, which is a reduction in platelet numbers in the blood, is a common *complication* of *chronic liver* disease. It increases the risk of *bleeding* during procedures including *liver* biopsy and *transplantation*. It can delay or prevent procedures, leading to illness and death. Established treatment largely involves platelet *transfusion* before the procedure or as rescue therapy for *bleeding*. This report aims to *systematically* review the clinical *effectiveness* and estimate the cost-*effectiveness* of the first two recently licensed treatments, thrombopoietin *receptor* agonists avatrombopag (Doptelet®; Dova *Pharmaceuticals*, Durham, NC, USA) (60 *mg* if platelet count is < 40,000/μl and 40 *mg* if platelet count is 40,000–< 50,000/μl) and lusutrombopag (Mulpleta ®; Shionogi *Inc*., London, UK) (3 *mg* if platelet count is < 50,000/μl), compared with established treatment.

From a comprehensive search, six studies were included. Clinical *effectiveness* analysis showed that avatrombopag and lusutrombopag were *superior* to no thrombopoietin *receptor* agonist in avoiding both platelet *transfusion* and rescue therapy. Only avatrombopag seemed *superior* to no thrombopoietin *receptor* agonist in reducing rescue therapy alone.

Cost-*effectiveness* analysis found that lusutrombopag and avatrombopag were more expensive than no thrombopoietin *receptor* agonist over a lifetime, as the savings from avoiding platelet *transfusions* were *exceeded* by the drug cost, and without long-term health benefits. The probabilistic *sensitivity* analysis, which examined the effect of uncertainty, showed that no thrombopoietin *receptor* agonist had 100% *probability* of being cost-effective. Uncertainty about the price of avatrombopag and the content and costs of platelet *transfusions* and the potential under-reporting of use to estimate platelet *transfusion*-specific *mortality* had the greatest impact on results. If the price of avatrombopag was (*confidential* information has been removed) below the price of lusutrombopag, avatrombopag would become cost saving in the 40,000–< 50,000/μl subgroup. However, although in some *scenarios* avatrombopag costs could *decrease* in the 40,000–< 50,000/μl subgroup to around 10% more than the cost of no thrombopoietin *receptor* agonist, there would be *negligible* health benefits and the *incremental* cost-*effectiveness ratios* would remain very high, meaning that lusutrombopag and avatrombopag would still not be considered cost-effective.

Armstrong N, Buyukkaramikli N, Penton H, Riemsma R, Wetzelaer P, Carrera VH, et al. Avatrombopag and lusutrombopag for thrombocytopenia in people with chronic liver disease needing an elective procedure: a systematic review and cost-effectiveness analysis. Health Technology Assessment 2020;24(51). <https://doi.org/10.3310/hta24510>
